# Serial Hemoglobin and Hematocrit Futility in Blunt Solid Organ Injury

**DOI:** 10.7759/cureus.118035

**Published:** 2026-10-16

**Authors:** Gunnar Orcutt, Casey Morehouse, Dominick Ricci, Jeremy Reeves

**Affiliations:** 1 Surgery, Prisma Health Midlands, University of South Carolina School of Medicine, Columbia, USA; 2 Trauma and Acute Care Surgery, Prisma Health Midlands, University of South Carolina School of Medicine, Columbia, USA

**Keywords:** blunt solid organ injury, futility, quality improvement, trauma, trauma outcomes

## Abstract

Introduction

Routine serial hemoglobin (Hgb) testing is commonly used in patients with blunt solid organ injury; however, the incremental predictive value of Hgb decline for subsequent operative (OR) or interventional radiology (IR) intervention remains uncertain. We evaluated whether a decline in Hgb was independently associated with intervention when considered alongside commonly assessed clinical and physiologic factors.

Methods

We retrospectively reviewed trauma patients with blunt liver, spleen, or kidney injuries admitted to a Level I trauma center (2021-2025). Out of 180 patients, those requiring immediate operation (n=103) were excluded. The remaining 77 patients were initially managed nonoperatively and later required OR or IR intervention. Candidate triggers included physical examination changes, hemodynamic changes, Hgb decline ≥2 g/dL, and trauma bay transfusion. Associations were analyzed using contingency tables and multivariable logistic regression.

Results

Physical examination changes, hemodynamic changes, and trauma bay transfusion were not associated with intervention within 24 h. An Hgb decline ≥2 g/dL was associated with lower odds of intervention (odds ratio (OR): 0.28, 95% confidence interval (CI): 0.08-1.00, p=0.049). Physiologic covariates did not improve model performance.

Conclusion

In this retrospective cohort, serial Hgb decline was not an independent predictor of OR or IR intervention. The inverse association observed should not be interpreted as evidence that Hgb monitoring has no clinical role. Rather, Hgb trends should be interpreted in conjunction with hemodynamic status, physical examination, imaging, transfusion requirements, and overall clinical trajectory.

## Introduction

Blunt solid organ injury involving the spleen, liver, or kidneys is a common consequence of traumatic injury and represents a significant source of morbidity in patients with trauma. Historically, many blunt solid organ injuries were managed operatively because of concerns about delayed clinical deterioration. Over the past several decades, however, the paradigm has shifted toward selective nonoperative management (NOM), driven by advances in computed tomography (CT) imaging, critical care monitoring, transfusion practices, and the expanding role of interventional radiology (IR) [1-3]. Consequently, most hemodynamically stable patients with blunt solid organ injury can be managed without surgery.

Current practice management guidelines from major trauma organizations emphasize physiologic stability and the patient's overall clinical condition when determining the candidacy for NOM [4]. Sustained hemodynamic stability or not, transfusion requirements, physical examination findings, and the presence or absence of peritonitis or other concerning clinical signs are central to decision-making. Injury grade and imaging findings remain important for risk stratification and may influence the need for angiographic or operative (OR) intervention; however, they should be interpreted in the context of the patient's overall clinical status.

Despite these advances, routine serial hemoglobin (Hgb) monitoring remains embedded in the management of patients with blunt solid organ injury. Serial Hgb measurements are frequently obtained at fixed intervals, often every six hours, with the intent of detecting occult hemorrhage and identifying patients at risk for clinical deterioration. The clinical utility of this practice remains uncertain because Hgb concentration may not immediately reflect acute blood loss and can be affected by crystalloid resuscitation, blood product administration, timing of blood draws, and physiologic equilibration.

Previous studies have suggested that decisions to pursue OR or IR intervention may be more strongly influenced by hemodynamic instability, imaging, and changes in clinical examination than by isolated laboratory trends [5,6]. Nevertheless, serial Hgb testing remains common and may contribute to repeated phlebotomy, patient discomfort, laboratory utilization, and healthcare costs.

The objective of this study was to determine whether a decline in Hgb independently predicts the need for OR or IR intervention among patients with blunt solid organ injury who were initially managed nonoperatively, when evaluated alongside other commonly assessed clinical and physiologic factors. Importantly, the study was not designed to determine whether Hgb monitoring should be abandoned or whether Hgb has no role in trauma management. Rather, we sought to determine whether serial Hgb decline provides independent predictive information that may assist in identifying failure of NOM in contemporary trauma practice.

## Materials and methods

Study design and patient selection

This study was reviewed and approved by the Institutional Review Board (IRB) of Prisma Health. A retrospective chart review was performed of adult patients with trauma admitted between January 2021 and December 2025 with blunt solid organ injury involving the liver, spleen, or kidneys who underwent OR or IR intervention during the index hospitalization.

Patients were identified using the institutional trauma registry data and subsequently verified through electronic medical record review. Inclusion criteria consisted of adult patients with blunt abdominal trauma and radiographically confirmed injury to the liver, spleen, or kidneys who subsequently underwent OR or IR intervention during the index hospitalization. Patients with penetrating trauma were excluded. A total of 180 patients met the initial inclusion criteria. Patients who underwent immediate OR intervention upon arrival, defined as direct transfer from the emergency department to the operating room, were excluded from the primary analysis because serial Hgb measurements would not reasonably be expected to influence management in this population. The final analytic cohort consisted of 77 patients who were initially managed nonoperatively and subsequently required OR or IR intervention.

Data collection and clinical variables

The retrospective nature of the study was acknowledged in the data abstraction process. Laboratory measurements, including serial Hgb values, were obtained from documented electronic medical record results. Clinical examination findings, hemodynamic changes, transfusion events, and indications for intervention were abstracted from contemporaneous clinical documentation. As documentation and timing of measurements were not prospectively standardized, the study is subject to potential misclassification and measurement-timing bias. These limitations were considered when interpreting associations between Hgb decline and intervention.

Demographic variables included age and sex. Injury-related variables included the injured organ, Injury Severity Score (ISS) [7], arterial blood gas (ABG) pH, base deficit, and administration of tranexamic acid (TXA). Candidate clinical triggers for intervention included new physical examination changes, a Hgb decline of ≥2 g/dL measured at approximately six-hour intervals, hemodynamic changes requiring intervention, and blood transfusion in the trauma bay. Trauma bay transfusion was further evaluated according to the number of units administered.

Study outcomes and statistical analysis

The primary outcome was OR or IR intervention within 24 hours of hospital arrival. Categorical variables were evaluated using chi-square or Fisher's exact tests, as appropriate. Associations between individual clinical triggers and the primary outcome were further evaluated using univariable binary logistic regression. Multivariable binary logistic regression was then used to evaluate whether candidate clinical triggers independently predicted intervention. Additional multivariable models incorporated physiologic and injury-severity variables, including ABG pH, base deficit, and ISS. Logistic regression results are reported as odds ratios (ORs) with corresponding 95% confidence intervals (CIs) and p-values. Statistical significance was defined as p<0.05.

Because intervention decisions in blunt solid organ injury are multifactorial, the analysis was intended to assess the predictive association of serial Hgb decline rather than to model Hgb as a stand-alone decision rule. Imaging findings, associated injuries, clinician judgment, and other factors that may influence intervention were not uniformly captured and therefore could not be incorporated comprehensively into the analysis.

## Results

Study cohort and baseline characteristics

The study cohort comprised 77 patients who were initially managed nonoperatively and later underwent OR or IR intervention. Patients who underwent immediate OR intervention were excluded from the primary comparative analysis.

Study groups were generally comparable with respect to demographic and injury-related characteristics. Mean age, sex distribution, and overall injury burden as measured by the ISS did not differ significantly between groups (all p>0.05). Similarly, there were no statistically significant differences in the distribution of solid organ injuries involving the spleen, liver, or kidneys.

Clinical trigger analysis and predictors of intervention

In the univariable analysis, changes in physical examination were not significantly associated with OR or IR intervention within 24 hours (odds ratio (OR): 1.67, 95% confidence interval (CI): 0.39-7.00, p=0.49). Also, hemodynamic changes were not significantly associated with the intervention (OR: 0.79, 95% CI: 0.20-3.00, p=0.73). Administration of TXA en route was not significantly associated with the intervention (OR: 0.52, 95% CI: 0.06-4.00, p=0.56). Similarly, trauma bay blood transfusion was not significantly associated with the intervention, including transfusion of one unit (OR: 2.28, 95% CI: 0.24-21.00, p=0.47) or two units (OR: 0.90, 95% CI: 0.20-4.00, p=0.89). Estimates for patients receiving four or eight units were highly unstable because of sparse observations, with ORs of approximately 4.6×10⁷ and 4.3×10⁷, respectively, and p=0.99 for both comparisons (Table 1, Figure 1).

**Table 1:** Odds ratios for candidate clinical triggers predicting operative or interventional radiology intervention within 24 hours. TXA: tranexamic acid

Trigger	Odds ratio (OR)	95% confidence interval (CI)	p-value	Interpretation
Physical exam change	1.67	0.39-7.0	0.49	Not significant
Hemoglobin drop ≥2 g/dL	0.29	0.08-1.0	0.049	Significant; inverse association
Hemodynamic change	0.79	0.20-3.0	0.73	Not significant
TXA given en route	0.52	0.06-4.0	0.56	Not significant
Trauma bay blood transfusion (one unit)	2.28	0.24-21.0	0.47	Not significant
Trauma bay blood transfusion (two units)	0.9	0.20-4.0	0.89	Not significant
Trauma bay blood transfusion (four units)	~4.6×10⁷	-	0.99	Very sparse; unreliable estimate
Trauma bay blood transfusion (eight units)	~4.3×10⁷	-	0.99	Very sparse; unreliable estimate

**Figure 1:**
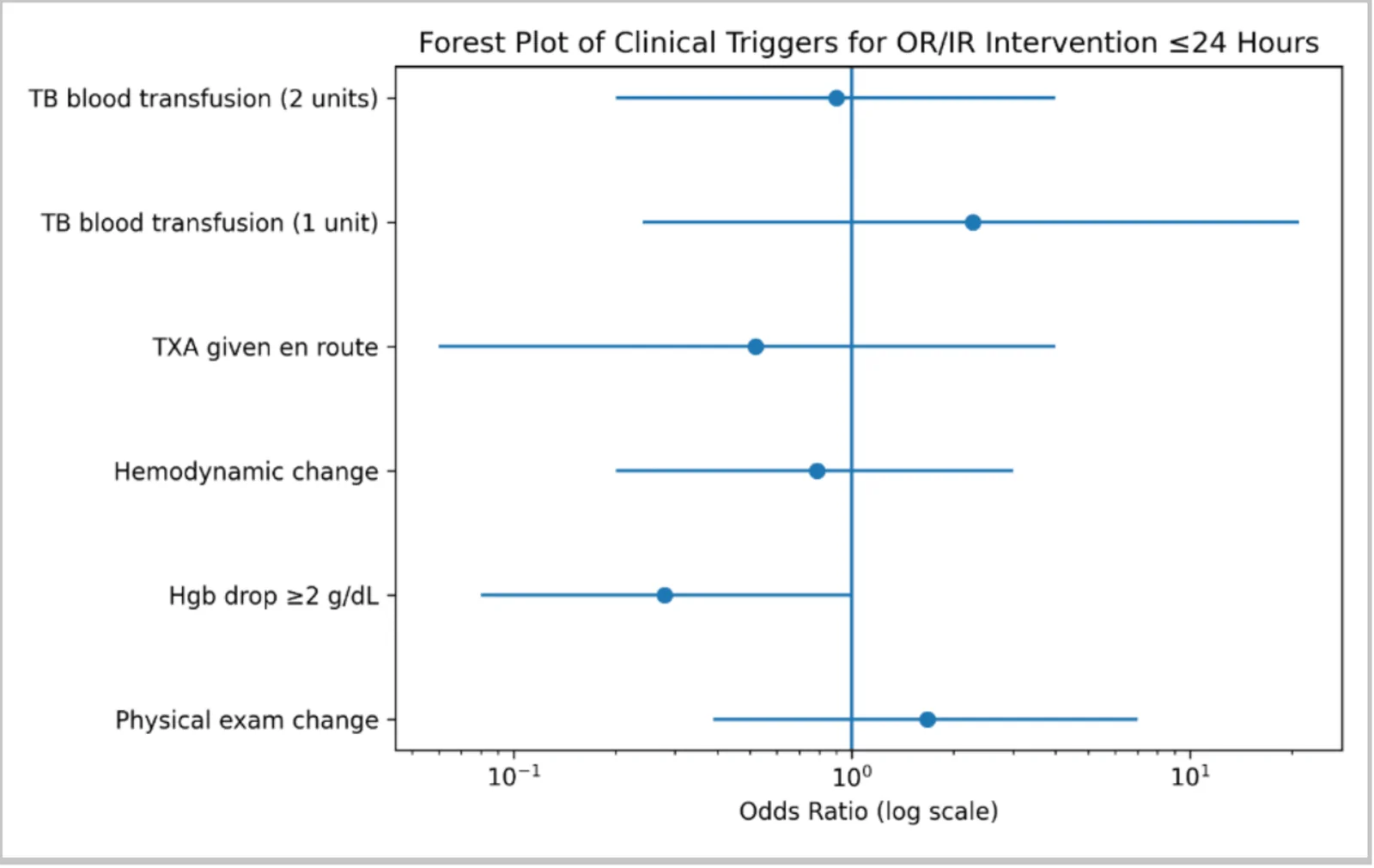
Forest plot demonstrating odds ratios and 95% confidence intervals for candidate clinical triggers associated with operative or interventional radiology intervention within 24 h of admission. The vertical reference line denotes an odds ratio of 1.0. JASP software (Jeffrey's Amazing Statistics Program; Department of Psychological Methods, University of Amsterdam, Amsterdam, the Netherlands) was used to create the figure. TXA: tranexamic acid; IR: interventional radiology; TB: trauma bay; Hgb: hemoglobin; OR: operative

An Hgb decline ≥2 g/dL was the only candidate clinical trigger demonstrating a statistically significant association with intervention. A Hgb decline of ≥2 g/dL was associated with lower odds of OR or IR intervention (OR: 0.29, 95% CI: 0.08-1.00, p=0.049). This inverse association should be interpreted cautiously and does not imply that Hgb decline protects against intervention. Rather, it may reflect differences in the timing of measurement relative to clinical decision-making or other unmeasured factors.

In multivariable logistic regression, none of the candidate clinical triggers independently predicted the need for OR or IR intervention within 24 hours. Addition of physiologic covariates, including ABG pH, base deficit, and ISS, did not meaningfully improve model performance. Overall model performance was poor, with reported Nagelkerke R² values <0.02, indicating that the variables included in the models explained little of the variability in intervention decisions.

## Discussion

This study evaluated whether serial Hgb decline independently predicts the need for OR or IR intervention in patients with blunt solid organ injury who were initially managed nonoperatively. The principal finding was that serial Hgb decline did not independently predict intervention in the multivariable analysis. Although a Hgb decline of ≥2 g/dL was associated with lower odds of intervention in univariable analysis, this inverse association should not be interpreted as evidence that Hgb decline is protective, that Hgb monitoring has no clinical role, or that Hgb should be excluded from trauma assessment. Instead, the finding suggests that Hgb decline alone may provide limited independent predictive information when determining whether a patient requires escalation of management.

Our findings should be interpreted within the broader framework of contemporary trauma care. The management of blunt solid organ injury is based on an integrated assessment that includes hemodynamic status, physical examination, transfusion requirements, injury characteristics, imaging findings, and overall clinical trajectory. The present study was not intended to evaluate any of these factors against Hgb in isolation or to suggest that serial Hgb testing should replace clinical assessment or imaging.

Our findings are consistent with those of previous studies questioning the incremental clinical utility of routine serial Hgb monitoring in blunt solid organ injury. Madbak et al. found that patients requiring urgent OR or angiographic intervention were identified based on hemodynamic changes and injury severity rather than changes in Hgb over time [5]. Similarly, Frotan et al. reported that most interventions occurred early after presentation and were driven primarily by hemodynamic instability and physical examination findings, with serial phlebotomy contributing little to subsequent management [6]. These findings are consistent with the current trauma guidelines, which emphasize hemodynamic stability, transfusion requirements, clinical examination, and overall clinical trajectory when determining whether NOM remains appropriate [1,8-10]. Our study adds to this literature by examining Hgb decline alongside other commonly considered clinical triggers in a contemporary Level I trauma population.

There are several reasons why Hgb trends may be an imperfect indicator of ongoing hemorrhage in this setting. Hgb concentration is an indirect and potentially delayed marker of blood loss and can be affected by crystalloid resuscitation, blood product administration, timing of blood draws, and physiologic equilibration after injury. A decline in Hgb, thus, does not necessarily indicate continued bleeding, particularly when it occurs without accompanying hemodynamic instability or changes in physical examination. Conversely, clinically significant hemorrhage may prompt intervention before a meaningful change in Hgb is observed. The decision to proceed with surgery or IR is therefore appropriately based on the patient's overall clinical trajectory rather than on an isolated laboratory value.

The inverse association between Hgb decline and the intervention in our cohort further illustrates the limitations of interpreting serial Hgb measurements outside their clinical context. This finding may reflect the differences in the timing of laboratory measurements relative to the intervention, treatment decisions made before subsequent Hgb results became available, resuscitation-associated hemodilution or hemoconcentration, or other clinical factors not captured in this retrospective analysis. In addition, Hgb decline may reflect bleeding from associated injuries, including mesenteric or retroperitoneal sources, rather than progression of the known solid organ injury. Failure of NOM is multifactorial, with injury characteristics, interval imaging findings, transfusion requirements, physiologic changes, physical examination, and clinician judgment contributing to the decision to intervene.

These considerations are particularly important when interpreting a retrospective study. Although the laboratory values are objectively recorded in the electronic medical record, Hgb measurement timing was not prospectively standardized, and clinical examination findings depend on the quality and completeness of contemporaneous documentation. Consequently, the present study can identify associations within the available cohort but cannot establish that serial Hgb decline causes, prevents, or independently determines intervention.

These findings may inform the ongoing discussion regarding routine fixed interval Hgb measurements in patients who remain clinically stable. Our results do not support eliminating Hgb monitoring. Rather, they suggest that the value of a serial Hgb result should be interpreted in relation to the patient's clinical condition and other indicators of potential deterioration. A selective approach to repeat Hgb measurement based on hemodynamic changes, physical examination findings, transfusion requirements, imaging concerns, or other clinical indications may warrant prospective evaluation.

This study has several limitations. Its retrospective, single-center design introduces the potential for selection bias, unmeasured confounding, and variation in provider decision-making. Although laboratory hemoglobin values were objectively documented in the electronic medical record, the timing of serial measurements was not prospectively standardized, and clinical examination findings were dependent on the completeness and accuracy of contemporaneous documentation. Furthermore, the decision to proceed with OR or IR intervention was multifactorial and may have been influenced by factors that were not comprehensively captured in the available dataset, including interval abdominal CT findings, changes in hemodynamics, clinician judgment, associated mesenteric or retroperitoneal injuries, and other sources of ongoing blood loss. Hemoconcentration and hemodilution following fluid or blood product resuscitation may also alter measured hemoglobin independently of ongoing hemorrhage. The relatively small sample size limits statistical power and may have contributed to imprecise estimates, particularly for less frequent clinical triggers. Finally, the findings should not be interpreted as demonstrating that hemoglobin monitoring has no clinical role or that intervention decisions can be based on hemoglobin trends alone. Rather, our findings suggest that serial hemoglobin decline did not provide independent predictive value for intervention within this retrospective cohort and should be interpreted alongside the patient's overall clinical condition and other available clinical and imaging information.

The paradoxical association between Hgb decline and intervention should therefore be interpreted cautiously and requires validation in larger prospective cohorts with standardized timing of laboratory measurements and systematic capture of hemodynamic, physical examination, transfusion, imaging, and associated injury variables. Future studies should evaluate whether a combination of these variables can more accurately identify patients at risk for failure of NOM.

## Conclusions

In this retrospective cohort of patients with blunt solid organ injury initially managed nonoperatively, serial Hgb decline did not independently predict the subsequent OR or IR intervention. The observed inverse association in univariable analysis should not be interpreted as evidence that Hgb monitoring has no clinical role. Rather, these findings suggest that Hgb trends should be interpreted within the broader clinical context, including hemodynamic status, physical examination, transfusion requirements, imaging findings, associated injuries, and overall clinical trajectory. Prospective multicenter studies are needed to determine when serial Hgb monitoring provides meaningful incremental information for identifying failure of nonoperative management.
